# Role of ghrelin in food reward: impact of ghrelin on sucrose self-administration and mesolimbic dopamine and acetylcholine receptor gene expression

**DOI:** 10.1111/j.1369-1600.2010.00294.x

**Published:** 2012-01

**Authors:** Karolina P Skibicka, Caroline Hansson, Emil Egecioglu, Suzanne L Dickson

**Affiliations:** Department of Physiology, Institute of Neuroscience and Physiology, The Sahlgrenska Academy at the University of GothenburgSweden

**Keywords:** Acetylcholine, dopamine, food motivation, ghrelin, GHS-R1A, operant conditioning

## Abstract

The decision to eat is strongly influenced by non-homeostatic factors such as food palatability. Indeed, the rewarding and motivational value of food can override homeostatic signals, leading to increased consumption and hence, obesity. Ghrelin, a gut-derived orexigenic hormone, has a prominent role in homeostatic feeding. Recently, however, it has emerged as a potent modulator of the mesolimbic dopaminergic reward pathway, suggesting a role for ghrelin in food reward. Here, we sought to determine whether ghrelin and its receptors are important for reinforcing motivation for natural sugar reward by examining the role of ghrelin receptor (GHS-R1A) stimulation and blockade for sucrose progressive ratio operant conditioning, a procedure used to measure motivational drive to obtain a reward. Peripherally and centrally administered ghrelin significantly increased operant responding and therefore, incentive motivation for sucrose. Utilizing the GHS-R1A antagonist JMV2959, we demonstrated that blockade of GHS-R1A signaling significantly decreased operant responding for sucrose. We further investigated ghrelin's effects on key mesolimbic reward nodes, the ventral tegmental area (VTA) and nucleus accumbens (NAcc), by evaluating the effects of chronic central ghrelin treatment on the expression of genes encoding major reward neurotransmitter receptors, namely dopamine and acetylcholine. Ghrelin treatment was associated with an increased dopamine receptor D5 and acetylcholine receptor nAChRβ2 gene expression in the VTA and decreased expression of D1, D3, D5 and nAChRα3 in the NAcc. Our data indicate that ghrelin plays an important role in motivation and reinforcement for sucrose and impacts on the expression of dopamine and acetylcholine encoding genes in the mesolimbic reward circuitry. These findings suggest that ghrelin antagonists have therapeutic potential for the treatment of obesity and to suppress the overconsumption of sweet food.

## INTRODUCTION

It is well-established that the circulating hormone ghrelin plays an important role in the regulation of energy balance (Kojima *et al*. 1999; Nogueiras, Tschöp & Zigman 2008). Released primarily by the stomach (Dornonville de la Cour *et al*. 2001), ghrelin elicits potent orexigenic effects both in rodents and in man (Wren *et al*. 2000, 2001) via stimulation of its central nervous system (CNS) receptor (Salomé*et al*. 2009a), the growth hormone secretagogue receptor (GHS-R1A) (Howard *et al*. 1996). Indeed, ghrelin targets hypothalamic and brain stem circuits involved in feeding and energy homeostasis (Dickson, Leng & Robinson 1993; Bailey *et al*. 2000; Hewson & Dickson 2000; Faulconbridge *et al*. 2003, 2008). Feeding behavior, however, is not only motivated by the need for nutrient repletion (i.e. the need to restore homeostasis); palatable high-fat and/or sugar foods can motivate intake despite a state of satiety (Zheng *et al*. 2009). The overconsumption of palatable natural reinforces such as sugar is a major factor driving the current obesity epidemic. It remains to be determined whether the central ghrelin signaling system is important for non-homeostatic sugar consumption, thereby providing a potentially important therapeutic target to suppress the intake of caloric, palatable and rewarding sweet foods.

Inspired by recent findings that ghrelin interacts with mesolimbic areas involved in non-homeostatic/reward feeding (Jerlhag *et al*. 2007), we sought to assess the role of ghrelin and its receptor in food motivation and goal-directed behavior for sucrose reward. These mesolimbic areas have long been the focus of drug addiction research as they are a major target for most drugs of abuse (Engel 1977; Koob 1992). The target mesolimbic pathway for ghrelin includes the dopamine projection from the ventral tegmental area (VTA) to the nucleus accumbens (NAcc) (Jerlhag *et al*. 2006, 2007), a pathway conferring reward from both addictive chemical drugs and natural rewards, including food (Koob 1992). Interestingly, GHS-R1A is expressed on dopaminergic neurons (Abizaid *et al*. 2006), implicating possible direct effects of ghrelin on the VTA dopamine system. These immunohistochemical data are complemented by the accumulating behavioral and electrophysiological evidence of ghrelin's effect in the VTA. For example, intra-VTA administration of ghrelin increases the activity of VTA dopamine neurons (Abizaid *et al*. 2006) and increases the release of dopamine into the NAcc (Jerlhag *et al*. 2007). Ghrelin also increases the activity of the cholinergic–dopaminergic link, an important reward pathway. Indeed, at least part of ghrelin's effects on dopamine seem to be mediated by the cholinergic system (Jerlhag *et al*. 2007).

While established that ghrelin has a potent orexigenic effect when food is readily available, it is not yet known whether the orexigenic effects of ghrelin can be extended to include changing motivation and reinforcing aspects of natural reinforces such as palatable sweet food (i.e. increasing wanting, and the effort/work one is willing to put into obtaining a sweet treat). The motivation and reward efficacy of drugs of addiction can be evaluated in the self-administration, operant conditioning model. Operant conditioning is a principal procedure for the analysis of motivated behavior that assesses acquired and voluntary behavior directed toward obtaining a reward. By measuring the amount of work a subject is willing to expend to obtain the reward, it offers an objective measure of reward value (Hodos 1961). Mesolimbic regions are crucial for motivational aspects of behavior including feeding and it is clear that ghrelin affects neuronal activity in relevant mesolimbic regions. What has not yet been shown is the direct effect of ghrelin on the motivation for high-sugar food. The primary aim of our study is to investigate whether the central ghrelin signaling system plays a role in the hedonic/motivational or positive reinforcing properties of high-sugar food reward and whether suppression of this system, utilizing a novel selective GHS-R1A antagonist JMV2959 (Salomé*et al*. 2009a), can suppress motivation to obtain sweets. GHS-R1A antagonists are currently being evaluated therapeutically in type 2 diabetic patients as suppression of ghrelin signaling has beneficial effects on glucose homeostasis (Sun *et al*. 2006), effects that would also benefit from reduced intake of sweet foods. Several lines of evidence suggest that dopaminergic and cholinergic neurotransmission play an important role in motivated reward behavior. Therefore, to further characterize the effects of ghrelin on central reward circuitry, we evaluated the impact of ghrelin treatment on dopamine and acetylcholine receptor gene expression changes in key reward nodes, the VTA and NAcc, after ghrelin treatment.

## METHODS

### Animals

Adult male Sprague-Dawley rats (200–250 g, Charles River, Germany) were housed in a 12-hour light/dark cycle with regular chow and water available *ad libitum*, except when indicated otherwise. All animal procedures were carried out with ethical permission and in accordance with the University of Gothenburg Institutional Animal Care and Use Committee guidelines.

### Surgery

For behavioral experiments targeting the CNS, a third ventricular guide cannula (26 gauge; Plastics One, Roanoke, VA, USA; coordinates: on the midline, 2 mm posterior to bregma, and 5.5 mm ventral to dura mater, with injector aimed 7.5 mm ventral to the dura) was implanted under isoflurane anesthesia. Cannulae were attached to the skull with dental acrylic and jeweler's screws and closed with an obturator, as described previously (Skibicka, Alhadeff & Grill 2009). Placement of the cannula in the third ventricle was verified one week after surgery by measurement of the sympathoadrenal-mediated glycemic response to central injection of 5-thio-D-glucose [210 µg in 2 µl of vehicle (saline)] (Ritter, Slusser & Stone 1981). In this placement verification protocol, a postinjection elevation of at least 100% of baseline plasma glucose level was required for subject inclusion. For the gene expression experiment, the rats were anesthetized (60–75 mg/kg Ketalar and 0.5 mg/kg Domitor i.p.; Pfizer, Sweden; Orion Co, Finland) and a chronic intracerebroventricular (ICV) cannula (Alzet Brain Infusion Kit II, DURECT Corp, Cupertino, CA, USA) was inserted into the lateral ventricle using the following coordinates: 0.6 mm posterior from bregma, 1.4 mm lateral from midline, 2.3 ventral from skull. The cannula was connected via a polyethylene catheter to an osmotic minipump (Alzet Mini-Osmotic Pump Model 2002, Durect, Cupertino, flow rate, 0.5 µl/hour for 14 days) implanted subcutaneously in the back of the animals.

### Operant conditioning model

#### Apparatus

Operant conditioning experiments took place in eight operant conditioning chambers designed for rats (30.5 × 24.1 × 21.0 cm; Medical-Associates, Georgia, VT, USA), which were placed in a sound-attenuated, dimly lit cabinet. Each chamber had a metal grid floor, two retractable levers with white light bulbs above them and a food pellet dispenser that can deliver 45 mg sucrose pellets (GlaxoSmithKline, Test Diet, Richmond, IN, USA) to the food tray. Data collection and processing were controlled by MED-PC software (Medical-Associates, Georgia, VT, USA).

#### Training

The procedure used for operant conditioning was adapted from (la Fleur *et al*. 2007) and (Tracy *et al*. 2008). All of the rats were subjected to a mild food restriction paradigm during which their initial body weight was gradually reduced to 90% over a period of one week. For the ICV-cannulated rats, the training commenced one week after the surgery. Prior to placement in the operant boxes, the rats were exposed to the sucrose pellets in the home cage environment on at least two occasions. Next, the rats learned to lever press for sucrose pellets under a fixed ratio FR1 schedule with two sessions per day. In FR1, a single press on the active lever resulted in the delivery of one sucrose pellet. All FR sessions lasted 30 minutes or until the rats earned 100 pellets, whichever occurred first. Most of the rats achieved the 100 pellets per session criterion after 10 to 15 sessions. Presses on the inactive lever were recorded but had no programmed consequence. FR1 schedule sessions were followed by FR3 and FR5 (i.e. three and five presses per pellet, respectively). Again, a minimum of 100 responses per session on the active lever was required for the advancement to the next schedule; most of the rats required only one to two FR3 and FR5 schedule(s) to achieve this level. The FR5 schedule was followed by the progressive ratio (PR) schedule during which the cost of a reward is progressively increased for each following reward in order to determine the amount of work the rat is willing to put into obtaining the reward. The response requirement increased according to the following equation: response ratio = [5e(0.2 × infusion number)] – 5 through the following series: 1, 2, 4, 9, 12, 15, 20, 25, 32, 40, 50, 62, 77, 95, 118, 145, 178, 219, 268, 328. The PR session ended when the rat had failed to earn a reward within 60 minutes. The break point was defined as the final completed ratio before the session ended. Responding was considered stable when the number of food pellets earned per session did not differ more than 15% for three consecutive sessions. In most cases, responding stabilized within five to seven sessions. PR test was carried out one session/day. The sessions lasted, on average, 75 minutes, although all the rats stayed in the operant boxes until 120 minutes to allow for all the sessions to end. The rats were subsequently transferred to home cages for a one hour free-feeding chow intake measurement. At the end of training and prior to testing, the rats were returned to an *ad libitum* feeding schedule.

#### Experimental design

All of the rats received intraperitoneal (IP) or in a separate group of rats, third ventricle (third ICV) injections early in the light cycle (for ghrelin tests) and late in the light cycle for ghrelin antagonist experiments 20 minutes prior to the start of operant testing. All conditions were separated by a minimum of 48 hours and run in a counterbalanced manner (each rat received all conditions on separate testing days).

##### Experiment 1: impact of peripheral or central ghrelin administration on PR operant responding for sucrose in rats

For all the rats, lever-pressing responses were examined after two conditions: IP treatment with saline or acylated rat ghrelin (Tocris, Bristol, UK; 0.33 mg/kg body weight at 1 ml/kg). The selected IP ghrelin dose has been shown previously to induce a feeding response in rats (Wren *et al*. 2000) and also to induce accumbal dopamine release and locomotor activity in mice (Jerlhag 2008). Subsequent to operant testing, the rats were allowed free access to chow, and chow intake was measured after a one-hour period. Next, in a separate group of rats, we examined responses after targeted CNS drug delivery after three conditions as follows: control condition with third ventricle saline, 0.5 µg or 1.0 µg of acylated rat ghrelin (Tocris) in a 1 µl volume. The selected doses of ghrelin has previously been shown to elicit feeding responses (Nakazato *et al*. 2001). For both the ICV and the IP ghrelin studies, lever-pressing experiments were performed in the satiated state (i.e. when food intake would be driven by the rewarding properties of the food rather than homeostatic drives). Also, in both studies, subsequent to operant testing, the rats were allowed free access to chow, and chow intake was measured after a one-hour period.

##### Experiment 2: impact of peripheral or central treatment with a ghrelin receptor (GHS-R1A) antagonist (JMV2959) on incentive motivation for a sucrose reward in rats

PR operant responses were examined after three conditions as follows: control condition with IP saline, 1 mg/kg or 3 mg/kg of JMV2959 (AEZS-123, AeternaZentaris GmBH, Frankfurt, Germany). The JMV2959 doses were selected based on Jerlhag *et al*. (2009) and Egecioglu *et al*. (2010) and preliminary data, previously shown to decrease conditioned place preference behavior but not have an independent effect on locomotor activity. Subsequent to operant testing, the rats were allowed free access to chow. To assess the effects of direct acute central antagonist action, in a separate group of rats, operant behavior was examined after the following three conditions: control condition with third ventricle saline injection, 5 µg or 10 µg of JMV2959 in a 1 µl volume. The selected ICV doses of JMV2959 dose was based on Salomé*et al*. (2009a) in which the orexigenic action of 1 µg ghrelin-administered ICV was blocked. Subsequent to operant testing, the rats were allowed free access to chow and chow intake was measured after a one-hour period and also at 24 hours after the initial injection. Studies with the GHS-R1A antagonist, in contrast to those performed with ghrelin (see earlier), were performed on the rats after a 16-hour food restriction prior to the injections in order to ensure high levels of endogenous circulating ghrelin (Cummings *et al*. 2001).

##### Experiment 3: ghrelin-induced changes in expression of dopamine- and acetylcholine-related genes in the VTA and NAcc

Here, we determined the effects of chronic ICV ghrelin infusion for two weeks on the expression of selected genes involved in dopaminergic and cholinergic transmission in two key mesolimbic reward pathway nodes, the VTA and NAcc. The selected dopamine-related genes were genes encoding the dopamine receptors (D1A, D2, D3, D5), catechol-O-methyltransferase, tyrosine hydroxylase (in VTA only) and monoamine oxidase A. The acetylcholine-related genes were: nicotinic receptor subunits (α3 α6, β2, β3). The genes we chose to evaluate have previously been implicated in ghrelin's effects and/or to reward/motivation behavior (Kelley *et al*. 2002; Figlewicz *et al*. 2006; Jerlhag *et al*. 2006, 2007; Sibilia *et al*. 2006; Dalley *et al*. 2007; Kuzmin *et al*. 2009; Lee *et al*. 2009; Nimitvilai & Brodie 2010; Perello *et al*. 2010). A chronic ghrelin/saline infusion protocol was used in preference to acute injection in order to increase chances of seeing an effect on gene expression; moreover, if ghrelin is an important regulator of the reward system in the long term, promoting overeating and obesity, its chronic effects to alter key reward mechanisms are likely to be of considerable importance.

#### Drug administration and tissue dissection

The catheter and the osmotic pump were filled with acetylated human ghrelin (gift from Rose Pharma, Copenhagen, Denmark) solution (8.3 µg/rat/day) or saline vehicle solution (0.9% NaCl); this dose and length of treatment has previously been show to affect gene expression in the hypothalamus (Salomé*et al*. 2009b). Fourteen days after implantation of the minipumps, the rats were killed by decapitation. The brains were rapidly removed and the VTA and the NAcc were dissected using a brain matrix (borders of each regions were determined based on Paxinos & Watson 1986), frozen in liquid nitrogen and stored at –80°C for later determination of mRNA expression.

#### RNA isolation and mRNA expression

Individual brain samples were homogenized in Qiazol (Qiagen, Hilden, Germany) using a TissueLyzer (Qiagen). Total RNA was extracted using RNeasy Lipid Tissue Mini Kit (Qiagen) or RNeasy Micro Kit (Qiagen), both with additional DNAse treatment (Qiagen). RNA quality and quantity were assessed by spectrophotometric measurements (Nanodrop 1000, NanoDrop Technologies, Wilmington, DE, USA). For cDNA synthesis, total RNA was reversed transcribed using random hexamers (Applied Biosystems, Sundbyberg, Sweden) and Superscript III reverse transcriptase (Invitrogen Life Technologies, Paisley, UK) according to the manufacturer's description. Recombinant RNaseout Ribonuclease Inhibitor (Invitrogen) was added to prevent RNase-mediated degradation. All the cDNA-reactions were run in triplicate. Real-time reverse transcription PCR was performed using TaqMan Custom Array assays. They were designed with TaqMan probe and primer sets for target genes chosen from an on-line catalogue (Applied Biosystems). Each port on the TaqMan Array platforms was loaded with cDNA corresponding to 100 ng total RNA combined with nuclease-free water and 50 µl TaqMan Gene Expression Master Mix (Applied Biosystems) to a final volume of 100 µl. The TaqMan Arrays were analyzed using the 7900HT system with a TaqMan Array Upgrade (Applied Biosystems). Thermal cycling conditions were: 50°C for two minutes, 94.5°C for 10 minutes, followed by 40 cycles of 97°C for 30 seconds and 59.7°C for one minute.

Gene expression values were calculated based on the ΔΔ*C*_t_ method (Livak & Schmittgen 2001), where the saline-treated group was designated the calibrator. Briefly, Δ*C*_t_ represents the threshold cycle (*C*_t_) of the target gene minus that of the reference gene and ΔΔ*C*_t_ represents the Δ*C*_t_ of the ghrelin treated group minus that of the calibrator. Relative quantities were determined using the equation relative quantity = 2^−ΔΔ*C*t^. For the calibrator sample, the equation is relative quantity = 2^−0^, which is 1; therefore, every other sample is expressed relative to this. Glyceraldehyde-3-phosphate dehydrogenase was used as reference gene.

### Statistics

All behavioral parameters were analyzed by analysis of variance followed by *post hoc* Tukey test or *t*-tests as appropriate. Statistical analyses were conducted using Statistica software (Tulsa, OK, USA). In order to analyze the effect of chronic central ghrelin treatment on gene expression, *t*-test was used, with *P*-values calculated using the Δ*Ct*-values. Differences were considered significant at *P* < 0.05. Data are expressed as mean ± SEM.

## RESULTS

### Experiment 1: impact of peripheral or central ghrelin administration on PR operant responding for sucrose in rats

**Figure 1:**
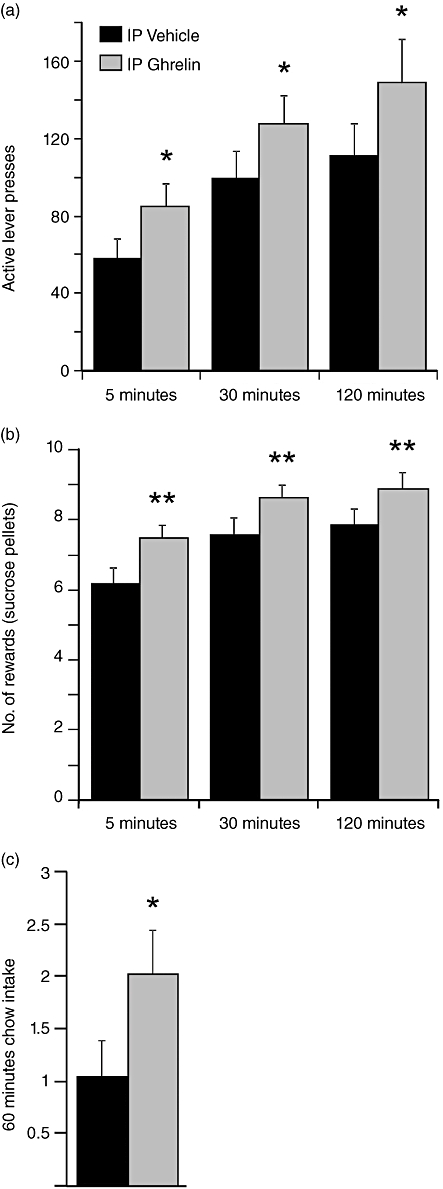
Peripheral ghrelin injection increases the motivation to obtain palatable food in a PR ratio operant conditioning model. The number of responses on the active lever (a) and the number of 45 mg sucrose rewards obtained (b) are significantly increased by 0.33 mg/kg IP ghrelin injection for a 120-minute period of operant testing. Intake of freely available chow is also increased by IP ghrelin injection (c). Data represent the mean ± SEM, *n* = 15, *P < 0.05, **P < 0.005 from vehicle

**Figure 2:**
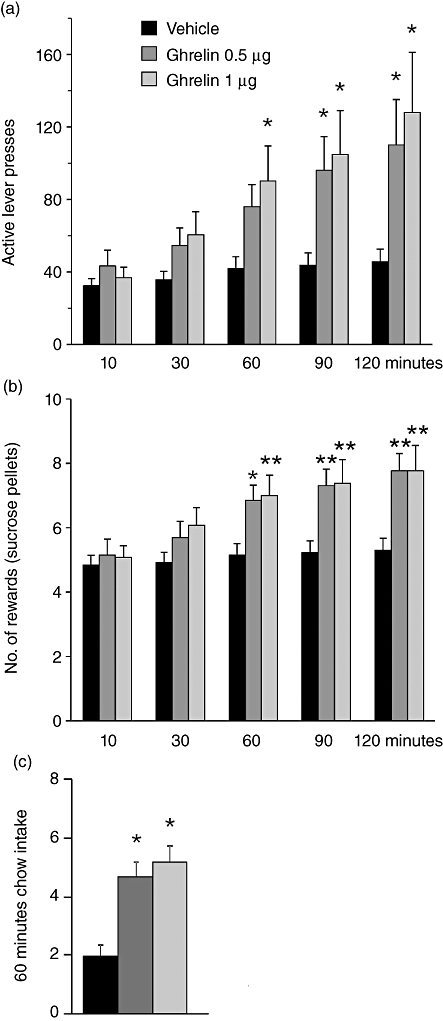
CNS (third ICV) ghrelin delivery increases the rewarding value of sucrose in a PR ratio operant conditioning model. The number of responses on the active lever (a) and the number of 45 mg sucrose rewards obtained (b) are significantly increased by third ICV ghrelin injection for the 120-minute period of operant testing. Short-term intake of freely available chow is also increased by IP ghrelin injection (c). Data represent the mean ± SEM, *n* = 13, *P < 0.05, **P < 0.005 from vehicle, *post hoc* Tukey analysis

Here, we employ a paradigm utilized in addiction research to assess the role of ghrelin in natural sweet food motivation and reinforcing properties of sugar. Specifically, to determine the role of peripheral ghrelin administration on sucrose reward efficacy, we examined sucrose self-administration in a progressive response schedule in the rats 20 minutes after IP injection of vehicle or ghrelin. All measures of operant behavior were significantly increased in the rats after acute peripheral ghrelin injection: active lever pressing (*P* < 0.05 for all time points), number of sugar pellets earned (*P* < 0.005 for all time points) and 120 minutes break point (*P* < 0.005, 32.53 ± 3.4 and 41 ± 4.3 for vehicle and ghrelin, respectively; Fig. 1a,b). The literature primarily supports a central site of action for ghrelin's orexigenic effect. However, GHS-R1A is also expressed outside of the CNS in sites relevant for food intake control, for example, on the vagus nerve; therefore, it can not be ruled out that part of the observed effects of IP ghrelin are mediated by those peripheral receptors. Central injection of a low volume and dose of ghrelin, however, stimulates only the CNS GHS-R1A. Therefore, in order to determine a direct CNS effect of ghrelin on sucrose reward efficacy, we performed a parallel study in which vehicle or ghrelin were administered by third ventricle injection, also 20 minutes prior to the operant paradigm. Consistent with a central site of effect hypothesis, acute ICV ghrelin injection to rats (both 0.5 µg and 1.0 µg doses) significantly increased all of the aforementioned measures of operant behavior (Fig. 2a,b). The time course of the active lever responses in the ICV ghrelin study revealed that while the effect emerged slowly during the 10- and 30-minute time points, it reached significance at 60 minutes [active lever: 10 minutes *F*(2, 24) = 0.94, *P* = 0.41, 30 minutes *F*(2, 24) = 3.13, *P* = 0.06, 60 minutes *F*(2, 24) = 5.86, *P* < 0.01, 90 minutes *F*(2, 24) = 6.42, *P* < 0.01, 120 minutes *F*(2, 24) = 6.03, *P* < 0.01; rewards earned: 10 minutes *F*(2, 24) = 0.26, *P* = 0.78, 30 minutes *F*(2, 24) = 2.76, *P* = 0.08, 60 minutes *F*(2, 24) = 8.31, *P* < 0.005, 90 minutes *F*(2, 24) = 10.16, *P* < 0.001, 120 minutes *F*(2, 24) = 11.93, *P* < 0.001; and break point: *F*(2, 24) = 7.22, *P* < 0.005 (17.31 ± 1.53, 33.15 ± 5.52, 36 ± 6.95 for vehicle, 0.5 µg and 1.0 µg ghrelin, respectively)], a time course consistent with other reports of ghrelin-induced feeding latency when delivered by this route (Faulconbridge *et al*. 2003). In both experiments, activity at the inactive lever was minor and did not differ significantly between the different treatment groups (IP 4.1 ± 1.1, 4.1 ± 1.1 for vehicle and ghrelin, respectively; ICV 3.9 ± 1.1, 2.1 ± 0.7, 3.5 ± 1.6 for vehicle, 0.5 µg and 1.0 µg ghrelin, respectively), suggesting that the treatment does not produce unspecific non-goal directed changes in activity. Immediately after operant testing, the rats were returned to their home cages and allowed free access to chow; the rats injected with ghrelin, whether given peripherally (*P* < 0.05) or centrally [*F*(2, 24) = 12.64, *P* < 0.001], nearly doubled their chow intake during the first hour as compared with the vehicle-treated groups (Figs 1c & 2c). In line with previous data (Faulconbridge *et al*. 2003) indicating that most of the hyperphagic effect of acute central ghrelin injection takes place within three hours after the injection, no effect on chow intake was noted in our study at three to 24 hours after ICV administration of either dose of ghrelin [17.4 ± 1.12, 18.42 ± 1.34, 19.12 ± 1.43 vehicle, 0.5 µg and 1.0 µg ghrelin, respectively, *F*(2, 24) = 2.27, *P* = 0.13].

### Experiment 2: impact of peripheral or central treatment with a ghrelin receptor (GHS-R1A) antagonist (JMV2959) on incentive motivation for sucrose reward in rats

**Figure 3:**
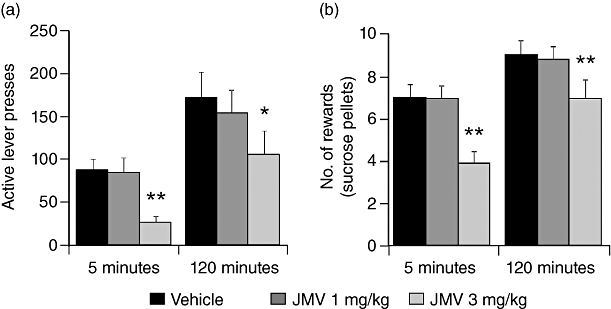
Peripheral delivery of a ghrelin receptor antagonist, JMV2959. decreases the motivation to obtain palatable food in a PR ratio operant conditioning model. The number of responses on the active lever (a) and the number of 45 mg sucrose rewards obtained (b) are significantly decreased by IP JMV2959 injection for the 120-minute period of operant testing. Data represent the mean ± SEM, *n* = 13, *P < 0.05, **P < 0.005 from vehicle, *post hoc* Tukey analysis

**Figure 4:**
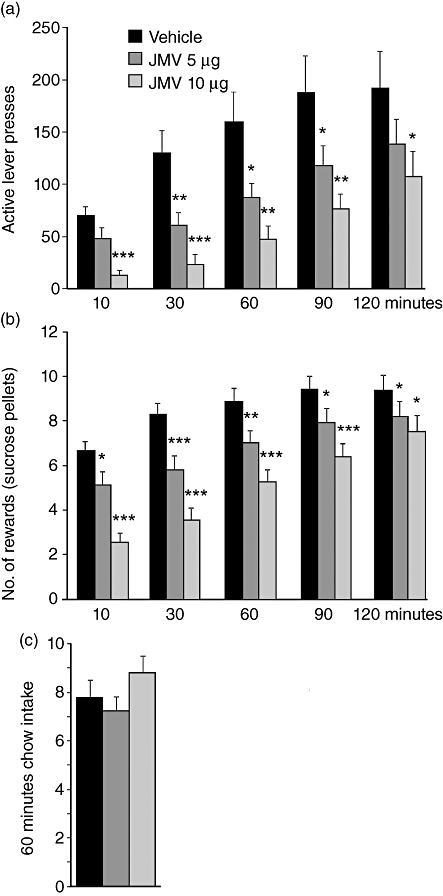
Central blockade of GHS-R1A with JMV2959 decreases the motivation to obtain food reward in a PR ratio operant conditioning model. The number of responses on the active lever (a) and the number of 45 mg sucrose rewards obtained (b) are significantly decreased by third ICV delivery of the GHS-R1A antagonist for the 120-minute period of operant testing. Short-term intake of freely available chow was not altered by central JMV2959 treatment in this paradigm (c). Data represent the mean ± SEM, *n* = 15, *P < 0.05, **P < 0.005, ***P < 0.0005 from vehicle, *post hoc* Tukey analysis

Next, we explored the effects of pharmacological blockade of GHS-R1A on sucrose reward efficacy. Thus, sucrose self-administration in a progressive response schedule was examined in the overnight food-restricted rats to ensure high levels of endogenous circulating ghrelin 20 minutes after IP injection of vehicle or 1 mg/kg or 3 mg/kg of JMV2959, a GHS-R1A antagonist. All of the measures of operant behavior were significantly decreased in the rats after peripheral injection of JMV2959 [active lever: five minutes *F*(2, 24) = 11.53 *P* < 0.0005, 120 minutes *F*(2, 24) = 11.27, *P* < 0.001; rewards earned: five minutes *F*(2, 24) = 23.39 *P* < 0.0005, 120 minutes *F*(2, 24) = 9.26, *P* < 0.001 and break point at 120: *F*(2, 24) = 5.98, *P* < 0.01 (45.31 ± 6.45, 42.08 ± 5.80, 30.0 ± 5.89 for vehicle, 1 mg/kg and 3 mg/kg JMV2959, respectively)]. *Post hoc* analysis revealed that the main effect was driven by the 3 mg/kg dose (Fig. 3a,b). To determine the role of the central ghrelin receptor in sucrose reward efficacy, a similar study was performed in which vehicle or JMV2959 (5 µg or 10 µg) was administered to the third ventricle 20 minutes before the operant measurements. All of the aforementioned measures of operant behavior were significantly decreased in the rats after acute third ventricle infusion of both doses of JMV2959 (Fig. 4a,b). The observed effect was immediate as *post hoc* analysis revealed significant differences among the treatment groups only after 10 minutes of activity in the operant chamber that were maintained throughout the testing period [active lever: 10 minutes *F*(2, 24) = 10.16, *P* < 0.0005, 30 minutes *F*(2, 24) = 11.48, *P* < 0.0005, 60 minutes *F*(2, 24) = 9.11, *P* < 0.001, 90 minutes *F*(2, 24) = 8.30, *P* < 0.001, 120 minutes *F*(2, 24) = 4.95, *P* < 0.05; rewards earned: 10 minutes *F*(2, 24) = 21.23, *P* < 0.0001, 30 minutes *F*(2, 24) = 25.08, *P* < 0.0001, 60 minutes *F*(2, 24) = 19.24, *P* < 0.0001, 90 minutes *F*(2, 24) = 20.04, *P* < 0.0001, 120 minutes *F*(2, 24) = 5.44, *P* < 0.01; and break point: *F*(2, 24) = 3.78, *P* < 0.05 (51.4 ± 8.58, 38.13 ± 5.07, 33.67 ± 5.21 for vehicle, 5 µg and 10 µg JMV2959, respectively)].

As expected (Hodos 1961; Jewett *et al*. 1995), in all the treatment groups, including both IP and ICV administration routes, the effect of food deprivation on the operant response for sucrose was evident (Figs 3a & 4a) and contrasts with that observed in the satiated state (Figs 1a & 2a). Activity on the inactive lever was minor (IP 9.6 ± 3.0, 6.8 ± 2.2, 5.6 ± 1.9 for vehicle and 1 mg/kg or 3 mg/kg JMV2959; ICV 6.4 ± 1.3, 4.6 ± 1.3, 4.4 ± 1.7 for vehicle, 5 µg and 10 µg of JMV2959, respectively) and whether administered peripherally or centrally, JMV2959 did not have any significant effect on that activity (this activity did not differ significantly between the different treatment groups). For the ICV study, immediately after the operant testing, the rats were returned to their home cages and allowed free access to chow; interestingly, no effect on chow intake was noted at either the one-hour (Fig. 4c) or 24-hour time point (data not shown). This could indicate that while ghrelin signaling is required for the deprivation-induced food motivation, it is not essential for the free feeding induced by 16 hours food deprivation likely because of other redundant mechanisms activated during a deprivation period. All of the free feeding measurements took place 140 minutes postinjection of the drug and so we can not exclude that the lack of effect is partially due to wash out of the drug.

### Experiment 3: ghrelin-induced changes in expression of dopamine- and acetylcholine-related genes in the VTA and NAcc

**Figure 5:**
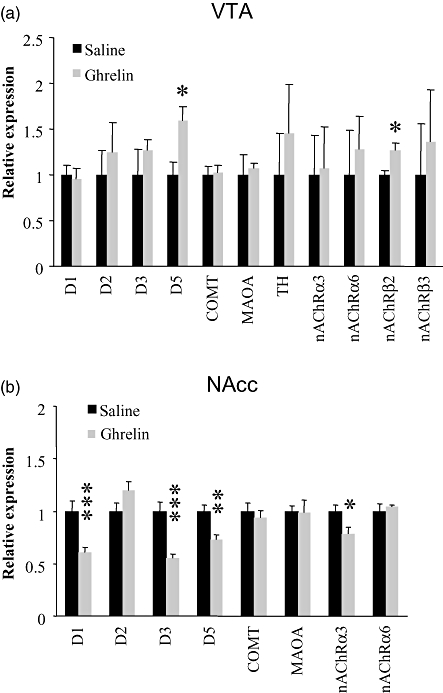
Dopamine and acetylcholine associated gene expression in VTA (a) and NAcc (b) after chronic ICV ghrelin or vehicle treatment. Data represent the mean of fold change relative to saline treatment. D1, dopamine D1 receptor; D2 dopamine D2 receptor; D3, dopamine D3 receptor; D5, dopamine D5 receptor; COMT, catechol-O-methyltransferase, TH tyrosine hydroxylase; and MAOA, monoamine oxidase A; nAChR, nicotinic acetylcholine receptor subunits α3 α6, β2, β3 *P < 0.05, **P < 0.005, ***P < 0.0005 from vehicle

In the present study, we also explored whether the dopamine- and acetylcholine-related genes are altered by ghrelin in key mesolimbic nodes, the VTA and NAcc, by examining the effects of chronic central ghrelin treatment on the expression of selected dopamine receptors and enzymes involved in dopamine production and metabolism, in a paradigm already established to produce ghrelin associated changes in gene expression in the hypothalamus (Salomé*et al*. 2009b). In the VTA dopamine receptor D5 and nicotinic acetylcholine receptor (nAChRβ2) had an increased mRNA expression in the ghrelin-treated rats compared with the saline-treated group (Fig. 5a). In the NAcc, there was a decreased mRNA expression of the genes encoding dopamine receptors D1A, D3 and D5 and also the nicotinic acetylcholine receptor nAChRα3 in the ghrelin-treated rats compared with the saline-treated group (Fig. 5b).

## DISCUSSION

Here, we reveal a role for the central ghrelin signaling system in the modulation of incentive motivation and reinforcing properties of sucrose reward and indicate an impact of chronic central ghrelin treatment on gene expression of dopaminergic and cholinergic receptors in key mesolimbic reward nodes. The results demonstrate that both central and peripheral delivery of ghrelin significantly increases the amount of work an animal is willing to do to obtain sucrose reward. Furthermore, systemic or central blockade of the ghrelin receptor suppressed operant responding for sucrose. Thus, we may infer that endogenous ghrelin signaling is of importance for the incentive motivation for a sucrose reward. Our findings are in line with the hypothesis that an important role of the central ghrelin signaling system is to increase the incentive value of rewards, including food. Given that food restriction increases the rewarding value of sucrose (Hodos 1961; Jewett *et al*. 1995) and that ghrelin levels are elevated during short-term food restriction (Gualillo *et al*. 2002), it is possible that during a state of food restriction/deprivation, ghrelin is one of the contributing factors that increases the rewarding value of food/food motivation. Indeed, peripheral ghrelin exposure increased operant behavior to levels similar to those observed in food-deprived rats, and conversely, blockade of ghrelin signaling decreased operant behavior to levels noted in the non-deprived rats.

It now seems clear that problematically increased food intake likely reflects a dysregulation of the central mechanisms of food reward, involving both hedonic and motivational aspects. As free feeding and reward-motivated feeding appear to be two separable phenomena with differential controlling neuroanatomical substrates (Salamone *et al*. 1991), it is important to examine both when assessing a role of agents involved in feeding behavior. Ghrelin's potent orexigenic effects have largely been studied in free feeding access models in which it would be difficult to distinguish between its role in nutrient repletion versus reward-motivated feeding. In the present study, we found that GHS-R1A ligands interfere with the motivation for sucrose reward, using an experimental model that has been used in other contexts to show wanting and motivation for addictive drugs of abuse. An increase in motivated behavior is common to both chemical drug addiction and caloric restriction and likely involves overlapping neurobiological mechanisms. In the present study, we also detected a ghrelin-induced increase in free feeding of normal chow food in the same animals that expended significantly more work for food in the operant chamber. Therefore, our data, taken together with earlier reports of ghrelin effects in free feeding models (Wren *et al*. 2000), indicate that ghrelin has the ability to modulate both free feeding as well as feeding motivation.

Given that the ghrelin receptor GHS-R1A is present in key hypothalamic, hindbrain and mesolimbic areas involved in energy balance and reward (Zigman *et al*. 2006) and that central ventricular injection of GHS-R1A ligands likely gain widespread access to these CNS areas, there could be several relevant neuroanatomical substrates for the sucrose reward motivation effect of ghrelin shown here. It seems likely that ghrelin acts directly on key mesolimbic areas as ghrelin activates VTA dopamine neurons (Abizaid *et al*. 2006) and direct administration of ghrelin to the VTA increases accumbal dopamine release (Jerlhag *et al*. 2007). Consistent with this, we have previously reported effects of intra-VTA ghrelin to increase the consumption of rewarding/palatable food in free choice feeding paradigms and also that lesions of the VTA blunt ghrelin-induced exploratory behavior of palatable food (Egecioglu *et al*. 2010). The NAcc may also be a direct target for ghrelin in modulating motivational aspects of food intake; when injected directly into this area, ghrelin induces a feeding response (Naleid *et al*. 2005), although the presence of GHS-R1A in this area in rodents was not described by other investigators (Zigman *et al*. 2006) and hence, requires further clarification.

Consistently, with its essential role in motivated behaviors, several genes within the dopamine system were altered by central ghrelin treatment. These data raise the possibility that regulation of dopamine receptor expression is a long-term mechanism via which ghrelin impacts on the reward-related function and signaling. Evaluation of dopamine receptors is not only important at the site of release such as the NAcc but also in the VTA as because of dendritic dopamine release (Cragg & Greenfield 1997), it is likely that it acts locally to influence reward motivated behaviors. Here, we found an increased expression of D5 in the VTA after ghrelin treatment. Dopamine D5 receptors are present on the cell bodies of dopaminergic VTA neurons (Ciliax *et al*. 2000) and their activity is required to restore the VTA dopamine neuron activity after a period of desensitization (Nimitvilai & Brodie 2010). In the NAcc, we noted a decreased expression of D1. In fact, reduced expression of this receptor has been recently shown in the NAcc of obesity prone but not obesity-resistant rats on high fat diet indicating its potential role in NAcc in obesity and overconsumption (Alsio *et al*. 2010). Also, the expression of genes encoding D3 was reduced by ghrelin, a finding of particular interest given the decreased availability of D2/D3 receptors in both rat and human drug users correlates with increased impulsivity (Dalley *et al*. 2007; Lee *et al*. 2009). Interestingly, we did not see any significant changes in the enzymes involved in dopamine synthesis or production.

The important role of the acetylcholine system for drug and food rewards is well-documented; here, we show that ghrelin treatment was associated with changes in expression of genes encoding several acetylcholine nicotinic receptors subunits, providing another route by which ghrelin can potentially alter reward function. Ghrelin can regulate VTA dopaminergic neurons indirectly via its action on the cholinergic neurons in the laterodorsal tegmental area (LDTg), an area rich in GHS-R1A, which is important for alcohol reward involving a cholinergic projection to the VTA dopamine system. In fact, previously, we showed that bilateral ghrelin injection into the LDTg in mice stimulates dopamine release in a cholinergic-dependent manner (Jerlhag *et al*. 2007, 2008) and increases consumption of alcohol in a free choice (alcohol/water) drinking paradigm (Jerlhag *et al*. 2009). Indeed, recent studies have implicated the cholinergic–dopamineric reward link in food reward (Dickson *et al*. 2010). Another interesting possibility is that ghrelin can enhance cholinergic signaling in the VTA via upregulation of cholinergic receptors. Indeed, our current gene expression data seems to support that mechanism as VTA nAChRβ2 mRNA levels were increased in the ghrelin-treated rats.

The function of NAcc cholinergic neurons and acetylcholine in the NAcc, on the other hand, have been more controversial with some reports indicating a role of acetylcholine in increasing reward-oriented behavior (Pratt & Kelley 2005; Pratt & Blackstone 2009) but others indicating that Ach in NAcc may act to inhibit feeding and play a role in satiety mechanism (Helm *et al*. 2003; Hoebel *et al*. 2007). Indeed, our results seem to be consistent with the latter as ghrelin treatment was associated with a decreased expression of one of the nicotinic receptor subunits, the nAChRα3. It is important to note that the gene expression studies, while very valuable in indicating potential downstream targets of ghrelin, only suggest the type of relationship (upregulation or downregulation) needed for expression of the orexigenic-/reward-oriented response but do not define it as it would be difficult to dissociate direct from compensatory changes. Therefore, our gene expression studies indicate a connection and provide a platform for future genetic and pharmacological studies determining the role of those genes in ghrelin's effects on free and reward-motivated feeding.

Although hypothalamic and brainstem areas most likely contribute to homeostatic feeding, we can not exclude an indirect role for hypothalamic and/or brainstem afferent systems in ghrelin-induced food reward motivation. Indeed, the orexinergic neurons project from the lateral hypothalamus to mesolimbic reward circuitry including the VTA and NAcc (Toshinai *et al*. 2003; Harris *et al*. 2005; Perello *et al*. 2010). The neuropeptide Y (NPY)/AgRP neurons of the arcuate nucleus, another target for GHS-R1A ligands (Dickson & Luckman 1997; Keen-Rhinehart & Bartness 2007a,b), may also play an important role. NPY has been shown to increase reward efficacy of chow as well as sucrose (Brown, Fletcher & Coscina 1998), whereas AgRP appears to increase reward efficacy of high-fat food only (Tracy *et al*. 2008). Ghrelin appears to have a role in both sucrose reward (present study) and in high-fat reward (Perello *et al*. 2010); however, the relative importance of the NPY/AgRP neurons for these effects of ghrelin remains to be elucidated. In summary, ghrelin has food motivational properties spanning across nutritional components and most likely affects several brain areas to synchronize a coordinated behavioral response to promote feeding.

Although ghrelin transport into the brain is limited (Banks *et al*. 2002), peripheral ghrelin appears to access and target areas such as the hippocampus (Diano *et al*. 2006) and VTA (Jerlhag 2008). Although there remains some debate over the relevance of the vagus nerve as an indirect route for ghrelin's central effects (Dornonville de la Cour *et al*. 2005; Date *et al*. 2002; Arnold *et al*. 2006), a direct action within the CNS seems likely as the effects of peripheral ghrelin on food intake can be suppressed by intra-VTA administration of ghrelin antagonists (Abizaid *et al*. 2006). Ghrelin is produced within the brain (Cowley *et al*. 2003), although it remains to be determined how this is regulated and whether brain-derived ghrelin provides an important centrally generated signal for food intake and for the motivation to eat. Taken together with the fact that the ghrelin receptor GHS-R1A is constitutively active (i.e. has activity in the absence of ghrelin ligand) (Holst *et al*. 2003), the question arises as to whether circulating ghrelin provides a physiologically relevant gut–brain signal for incentive motivation for food reward. The results of the present study, showing similar effects on sucrose reward work can be obtained via central and peripheral administration of GHS-R1A ligands, could indicate that both centrally released as well as peripherally released ghrelin can potentially affect food motivation.

In conclusion, our new data provide new evidence that ghrelin signaling is important for the motivation to obtain sucrose reward and impact on dopaminergic and cholinergic gene expression in mesolimbic reward pathway. Our findings inspire important questions regarding the role of the endogenous ghrelin in determining the incentive value for natural rewards such as sugar, in normal appetitive behavior and in the pathophysiology of eating disorders and obesity. Although significant work remains to relate causally the molecular changes in the dopamine and acetylcholine system to impact of ghrelin on reward, our data potentially indicate a novel mechanism by which ghrelin impacts on the reward behavior. Understanding ghrelin's role in reward processes is important for the understanding of the overlapping neurobiology of eating disorders and chemical drug addiction and provides a potential avenue for understanding the etiology of these diseases and for the development of novel therapies. Finally, the possibility to suppress problematic overeating of palatable sweet foods using GHS-R1A antagonists may have clinical and therapeutic relevance for the emerging beneficial effects of such compounds for blood glucose control (Sun *et al*. 2006) in type 2 diabetic patients (Esler *et al*. 2007).
